# Lung Ultrasound as a First-Line Test in the Evaluation of Post-COVID-19 Pulmonary Sequelae

**DOI:** 10.3389/fmed.2021.815732

**Published:** 2022-01-13

**Authors:** David Clofent, Eva Polverino, Almudena Felipe, Galo Granados, Marta Arjona-Peris, Jordi Andreu, Ana L. Sánchez-Martínez, Diego Varona, Laura Cabanzo, Jose M. Escudero, Antonio Álvarez, Karina Loor, Xavier Muñoz, Mario Culebras

**Affiliations:** ^1^Department of Respiratory Medicine, Vall D'Hebron University Hospital, Barcelona, Spain; ^2^Vall D'Hebron Institut de Recerca (VHIR), Barcelona, Spain; ^3^Radiology Department, Vall D'Hebron University Hospital, Barcelona, Spain; ^4^CIBER Enfermedades Respiratorias (CIBERES), Barcelona, Spain

**Keywords:** COVID-19, SARS-CoV-2, lung ultrasound (LUS), ultrasonography, pulmonary sequelae, interstitial lung disease (ILD)

## Abstract

**Background:** Interstitial lung sequelae are increasingly being reported in survivors of COVID-19 pneumonia. An early detection of these lesions may help prevent the development of irreversible lung fibrosis. Lung ultrasound (LUS) has shown high diagnostic accuracy in interstitial lung disease (ILD) and could likely be used as a first-line test for post-COVID-19 lung sequelae.

**Methods:** Single-center observational prospective study. Follow-up assessments of consecutive patients hospitalized for COVID-19 pneumonia were conducted 2–5 months after the hospitalization. All patients underwent pulmonary function tests (PFTs), high-resolution computed tomography (HRCT), and LUS. Radiological alterations in HRCT were quantified using the Warrick score. The LUS score was obtained by evaluating the presence of pathological B-lines in 12 thoracic areas (range, 0–12). The correlation between the LUS and Warrick scores was analyzed.

**Results:** Three hundred and fifty-two patients who recovered from COVID-19 pneumonia were recruited between July and September 2020. At follow-up, dyspnea was the most frequent symptom (69.3%). FVC and DLCO alterations were present in 79 (22.4%) and 234 (66.5%) patients, respectively. HRCT showed relevant interstitial lung sequelae (RILS) in 154 (43.8%) patients (Warrick score ≥ 7). The LUS score was strongly correlated with the HRCT Warrick score (r = 0.77) and showed a moderate inverse correlation with DLCO (r = −0.55). The ROC curve analysis revealed that a LUS score ≥ 3 indicated an excellent ability to discriminate patients with RILS (sensitivity, 94.2%; specificity, 81.8%; negative predictive value, 94.7%).

**Conclusions:** LUS could be implemented as a first-line procedure in the evaluation of Post-COVID-19 interstitial lung sequelae. A normal LUS examination rules out the presence of these sequelae in COVID-19 survivors, avoiding the need for additional diagnostic tests such as HRCT.

## Introduction

The global coronavirus disease 2019 (COVID-19) pandemic has reached unprecedented dimensions, with more than 246 million cases and 5 million deaths worldwide (1). Multiple studies published over the last year have described the pathogenesis and clinical characteristics of this disease (2, 3); however, the long-term sequelae of COVID-19 remain uncertain (4).

In previous viral epidemics such as those caused by MERS-CoV and SARS-CoV, a variable percentage of survivors developed interstitial lung disease (ILD), including pulmonary fibrosis (PF) (5–9). On a similar note, several recent reports have described early respiratory sequelae following COVID-19, such as persistent symptoms, impaired pulmonary function, and interstitial lung abnormalities (10–16). The histological findings in lung biopsies of these patients seem to be suggestive of organizing pneumonia and pneumonitis in a recent study (17). However, the magnitude, severity, and reversibility of these sequelae remain to be defined, and close follow-up after COVID-19 pneumonia is needed. Although different follow-up protocols have been published so far, the proposed diagnostic procedures are quite heterogeneous (18–23). For example, while chest imaging is always recommended in the initial evaluation, there is no consensus on the differential use of chest radiography (CXR) vs. computed tomography (CT). Interestingly, lung ultrasound (LUS) has not yet been considered in any of these protocols.

In the last decades, LUS has proven to be a suitable alternative to conventional radiological methods in multiple kinds of lung diseases (24–27). During the current pandemic, numerous studies have reported the usefulness of LUS as a front-line tool in the diagnosis and management of SARS-CoV-2 acute pneumonia (28–31). However, its applicability in the detection of post-COVID-19 interstitial sequelae is yet to be defined.

LUS has been previously validated for the detection of ILD secondary to other causes. Several studies, mostly those involving connective tissue disease-associated ILD, have shown significant superiority of LUS over CXR and, most importantly, similar sensitivity and negative predictive value in comparison with CT (32–37). In addition, ultrasonography can easily be considered a first-line tool because it is non-invasive, non-ionizing, and inexpensive.

Thus, the aim of this study was to describe the diagnostic accuracy of LUS in the assessment of early interstitial sequelae after COVID-19 pneumonia in comparison with CT.

## Methods

### Ethics Statement

Ethics approval was obtained prior to the start of the study by the Medical Ethics Committee of Vall d'Hebron Barcelona University Hospital [PR(AG)461/2020]. Written informed consent was obtained from all the patients before their inclusion.

### Study Design and Participants

This single-center observational prospective study evaluated 362 survivors hospitalized for COVID-19 pneumonia between March 3, 2020 and April 29, 2020 at Vall d'Hebron University Hospital, Barcelona, Spain. Patients were included consecutively if they visited the dedicated post-COVID-19 outpatient clinic at our respiratory department between July 20, 2020, and September 21, 2020.

Patients visited the clinic at least 2 months after their hospital discharge. Patients recovered from severe pneumonia were visited first for medical reasons. We excluded patients with previously diagnosed ILD, congestive heart failure and those who declined to participate. Baseline information was retrieved from medical records. All patients were interviewed face-to-face by experienced pulmonologists and underwent pulmonary function tests (PFTs), including spirometry and measurement of the carbon monoxide diffusing capacity (DLCO) and high-resolution CT (HRCT) and LUS. HRCT and LUS were performed no more than 15 days apart.

### Pulmonary Function Tests

Lung-function tests were performed at our dedicated laboratory by using Master-Lab equipment (E. Jaeger, Germany) in accordance with international protocols and GLI reference values (38–40). Pulmonary parameters included forced vital capacity (FVC), forced expiratory volume in the first second (FEV_1_), FEV1/FVC ratio, and lung diffusing capacity for carbon monoxide (DLCO). Lung volumes and post-bronchodilation tests were not performed.

### Chest HRCT

HRCT examinations were performed by a standard protocol using Siemens Somatom Force Dual Source CT and Siemens Somatom Definition AS+ scanners. Scans were obtained at full inspiration from the apex to the lung base in the supine position. All chest CT scans were reconstructed with a 1.5-mm slice thickness. Iodine contrast agents were not used.

HRCT images were evaluated according to the Fleischner Society glossary (41), and the following findings were evaluated: ground-glass opacities (GGO), consolidation, septal/subpleural lines (including parenchymal bands and reticular pattern), irregular pleura, nodules, subpleural cysts, architectural distortions, traction bronchiectasis, honeycombing, atelectasis, pleural effusion, and mosaic attenuation patterns.

Pulmonary involvement was quantified according to the Warrick score, which has been previously validated in scleroderma-related ILD (42, 43) and is widely used in ultrasonography studies (32–35). This score is obtained by summing the scores for five basic radiological ILD findings (from 0 to 5) and the extent of these changes (from 0 to 3). The total score ranges from 0 to 30, and a minimum score of 7 has been validated as the best cut-off point for predicting pulmonary disease in ILD (43). We implemented this cut-off point in our population and therefore classified patients with relevant interstitial lung sequelae (RILS) as those with a Warrick score ≥ 7. A more detailed explanation of the Warrick score is provided in Table 1.

**Table 1:** Warrick score for HRCT involvement^*^.

	**Point value**
**HRCT abnormality** ^a^	
Ground-glass opacities	1
Irregular pleura	2
Septal/subpleural lines	3
Honeycombing	4
Subpleural cysts	5
**Number of involved bronchopulmonary segments** ^b^	
1–3	1
4–9	2
>9	3

* *Adapted from Warrick et al. (42)*.

a *Each abnormality in HRCT is assigned a point value (maximum score is 15 if all abnormalities are present)*.

b *Disease extension is determined by counting the number of bronchopulmonary segments involved in each abnormality (total score, 15 points). The total score is calculated by summing the scores of the five basic HRCT abnormalities and disease extension, ranging from 0 to 30*.

Each HRCT image was evaluated by two blinded, independent thoracic radiologists who calculated the Warrick score. The final Warrick score was the average of the two independent reads if the difference was 4 points or less (variability < 13.3%). In cases with a greater difference, both radiologists met and performed a third consensual assessment.

### Lung Ultrasound

LUS examinations were performed using a Sonosite M-Turbo system equipped with a 2–5-MHz convex transducer. A team of four pulmonologist with expertise in LUS conducted all the examinations. Every patient underwent LUS at the follow-up visit by one of the four examiners mentioned, who was always in a separate consultation, blinded to the clinical and other exploration findings.

**Figure 1:**
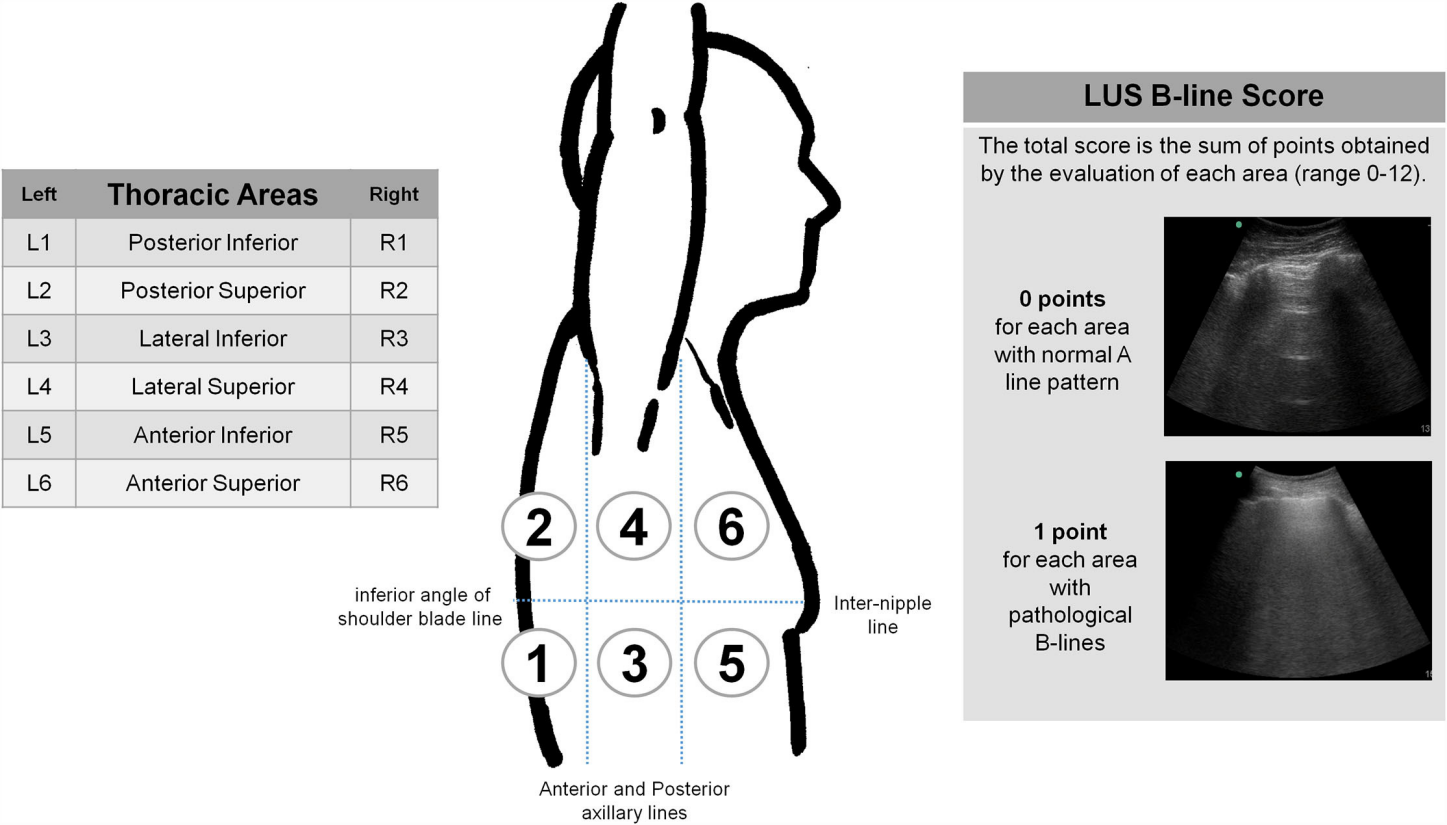
Thoracic areas for LUS examination protocol. Anatomical landmarks: Anterior and posterior axillary lines (for anterior, lateral and posterior areas). Inter-nipple line and inferior angle of shoulder blade line (for superior and inferior areas). Sternum and vertebral spine (for right and left sides).

**Figure 2:**
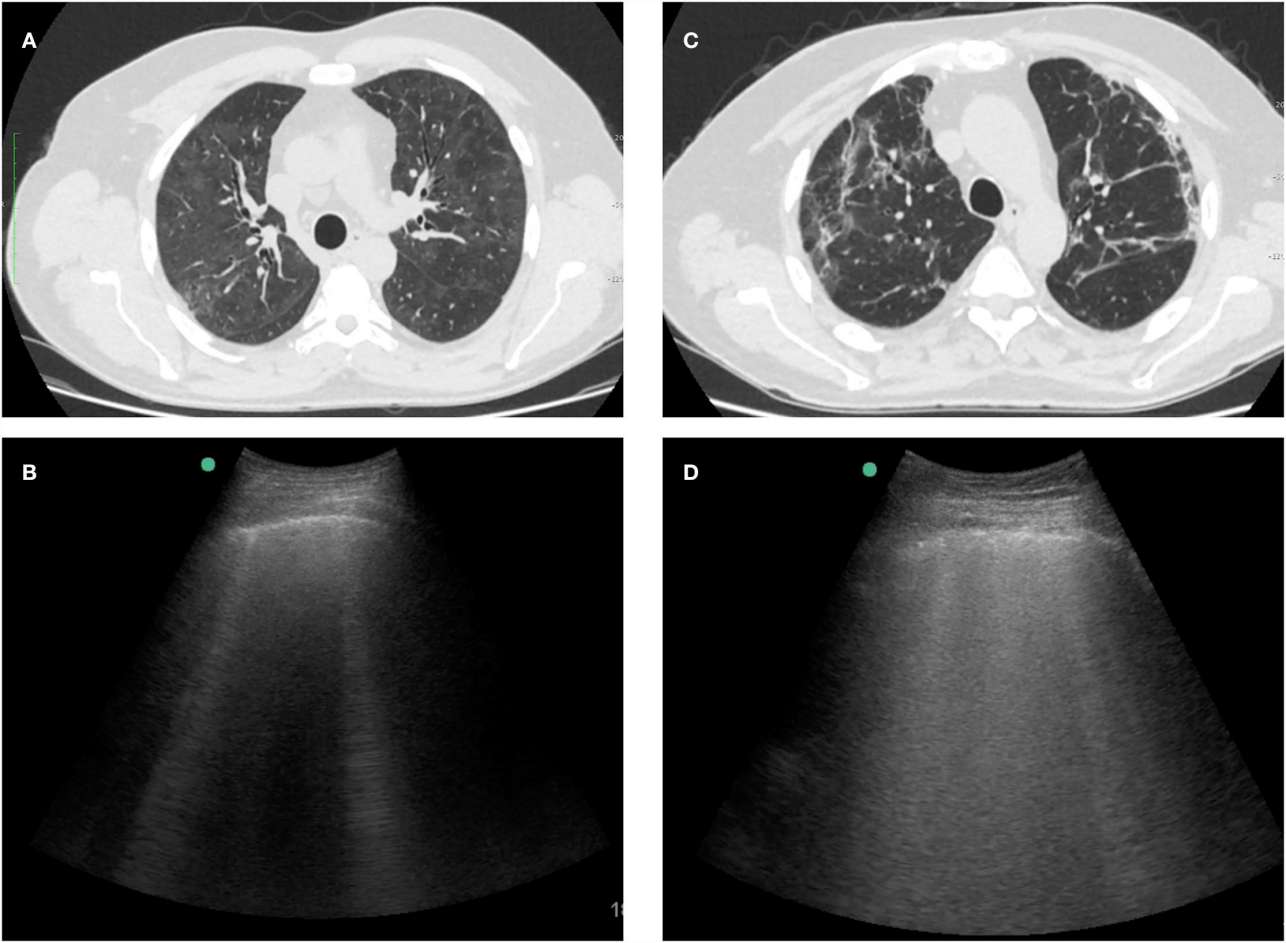
Examples of HRCT and LUS images from 2 patients with interstitial lung sequelae after COVID-19. **(A)** Persistent diffuse ground-glass opacities on HRCT. **(B)** LUS of the same patient with isolated B-lines and absence of pleural line abnormalities. **(C)** Subpleural lines and parenchymal bands on HRCT. **(D)** LUS of the same patient with confluent B-lines and blurred pleural line.

The scan protocol consisted of a complete examination of all intercostal spaces, which were divided into 12 thoracic areas (two posterior, two lateral, and two anterior for each side) (Figure 1). Abnormalities such as pleural line alterations, B-line artifacts, pleural effusion, and consolidations were registered for each thoracic area (Figure 2). B-lines were defined as laser-like vertical hyperechoic reverberation artifacts that arise from the pleural line extending to the bottom of the screen, moving synchronously with lung sliding (44). Each thoracic area was considered pathological when three or more B-lines were present in any intercostal space (44). We developed a B-line score by summing 1 point for each thoracic area with pathological B-lines (score range, 0 to 12). The inter-observer agreement was evaluated retrospectively by a blinded and simultaneous review of 250 saved clips by the four examiners.

### Outcomes

Primary endpoint was to establish the accuracy of LUS in the detection of relevant interstitial lung sequelae (RILS) in COVID-19 survivors in comparison with HRCT.

### Statistical Analysis

All data were analyzed using Stata software (StataCorp. 2017; *Stata statistical software: Release 15* College Station, TX, StataCorp LLC, USA). Descriptive analysis was performed according to the pneumonia severity groups and the presence of RILS at follow-up. For the qualitative variables, frequencies and percentages were compared using the chi-square test or Fisher's exact test. For the quantitative variables, means (SD) and medians (IQR) were calculated and compared using the Mann-Whitney U-test. The normality of the distribution was analyzed using the Shapiro–Wilk test. The *p*-value was computed from the Spearman rank correlation coefficient when the variable was continuous and from the χ^2^ test if the variable was categorical. Statistical significance was set at *p* < 0.05.

The sample size was not calculated because of the lack of reports defining the frequency of pulmonary sequelae in COVID-19 survivors when our study was designed.

LUS inter-observer agreement was evaluated for 250 measurements by using a contingency table and calculating the kappa index of agreement.

A Pearson correlation analysis was performed between the LUS score, HRCT Warrick score, and PFT variables.

Receiver operating characteristic (ROC) curves were plotted to analyze area under the curves (AUC). A cut-off point in the LUS score was obtained according to Youden index as the value corresponding to a HRCT Warrick score of ≥7.

## Results

### Study Population

Three-hundred and sixty-two patients who had recovered from COVID-19 pneumonia after hospitalization were evaluated. Patients with previously diagnosed ILD (*n* = 3), congestive heart failure (*n* = 2) and those who declined to participate (*n* = 5) were excluded. Consequently, 352 patients were included in our study. Their demographic and clinical characteristics are presented in Table 2. The median duration (IQR) of hospitalization was 9.5 [6.0–21.0] days, and 115 (32.7%) patients were admitted to the intensive care unit (ICU), with a median hospitalization period of 12 (6–21) days. Eighty-one patients (23.0%) required IMV, 51 (14.5%) required NIMV or HFNC, 109 (31.0%) required LFO, and 111 (31.5%) had mild pneumonia with no oxygen therapy requirement.

**Table 2:** Baseline characteristics of the study population.

**Characteristics**	**Total (*N* = 352)**
**Age, years, median (IQR)**	56 (48–67)
**Sex, *n* (%)**	
Men	203 (57.7)
**Smoking, *n* (%)**	
Never-smokers	249 (70.7)
**Comorbidities, *n* (%)**	
Hypertension	126 (35.8)
Diabetes	55 (15.6)
Dyslipidemia	79 (22.4)
Congestive heart failure	5 (1.4)
Chronic renal disease	25 (7.1)
BMI 25–29.9	148 (42.0)
BMI > 30	119 (33.8)
COPD	22 (6.3)
Asthma	4 (1.1)
**Severity groups, *n* (%)**	
Group 1, mild pneumonia: no oxygen requirement	111 (31.5)
Group 2, moderate pneumonia: LFO	109 (31.0)
Group 3, severe pneumonia: HFNC or NIMV	51 (14.5)
Group 4, critical pneumonia: IMV	81 (23.0)
**Respiratory complications, *n* (%)**	
Pulmonary embolism	15 (4.3)
Hemoptysis	4 (1.1)
Pneumothorax	1 (0.3)

*BMI, body mass index; COPD, chronic obstructive pulmonary disease; HFO, high-flow oxygen; ICU, intensive care unit; IMV, invasive mechanical ventilation; IQR, interquartile range; LFO, low-flow oxygen; NIMV, non-invasive mechanical ventilation*.

### Symptoms, PFT, HRCT at Follow-Up

The follow-up characteristics of the included patients are shown in Tables 3, 4. The median time (IQR) from hospital admission to the follow-up visit was 90 (64.0–114.0) days. Two hundred and forty-four (69.3%) patients had some persistent symptoms, with the most frequent symptoms being dyspnea (48.3%), fatigue (36.6%), and myalgia or arthralgia (24.1%). In assessments of pulmonary function, FVC and DLCO alterations (<80% of the lower limit range) were present in 79 (22.4%) and 234 (66.5%) patients, respectively. HRCT abnormalities at follow-up were also observed. Ground-glass opacities, consolidations, septal and subpleural lines, and mosaic attenuation patterns were observed in 244 (69.3%), 33 (9.4%), 229 (65.1%), and 88 (25%) patients, respectively. Fibrotic changes such as architectural distortion and traction bronchiectasis were found in 47 (13.4%) and 26 (7.4%) patients, respectively. Only 18 (5.1%) patients had subpleural cysts, and 4 (1.1%) had honeycomb cysts. When the Warrick score was calculated, 154 (43.8%) patients had a score ≥ 7 (RILS group), and 198 (56.2%) had a score < 7. None of the patients with a Warrick score below 7 showed any fibrotic alterations.

**Table 3:** Persistent symptoms at follow-up in relevant interstitial lung sequelae (RILS) groups classified by the Warrick score.

	**Non-RILS group (*n* = 198)**	**RILS group (*n* = 154)**	**Total (*N* = 352)**	^*^P-value
**Persistent symptoms, *n* (%)**				
Any symptoms	126 (63.6)	118 (76.6)	244 (69.3)	0.010
Dyspnea mMRC score = 0	121 (61.1)	61 (39.6)	182 (51.7)	<0.001
Dyspnea mMRC score ≥ 1	77 (38.9)	93 (60.4)	170 (48.3)	
Cough	34 (17.2)	23 (14.9)	57 (16.2)	0.558
Expectoration	5 (2.5)	0 (0)	5 (1.4)	0.071
Chest pain	22 (11.1)	15 (9.7)	37 (10.5)	0.677
Fatigue or muscle weakness	64 (32.3)	65 (42.2)	129 (36.6)	0.056
Myalgia or arthralgia	44 (22.2)	41 (26.6)	85 (24.1)	0.339
Headache	13 (6.6)	4 (2.6)	17 (4.8)	0.085
Sleep difficulties	9 (4.5)	3 (1.9)	12 (3.4)	0.186
Fever	0 (0.0)	3 (1.9)	3 (0.9)	0.083
Digestive symptoms	4 (2.0)	2 (1.3)	6 (1.7)	0.699
Taste or smell disorder	8 (4.0)	7 (4.5)	15 (4.3)	0.816

* *P-value comparisons between non-RILS and RILS groups*.

*RILS, relevant interstitial lung sequelae, defined by a Warrick score ≥ 7*.

*IQR, interquartile range; MMRC, modified medical research council; RILS, relevant interstitial lung sequelae*.

**Table 4:** PFT and HRCT findings at follow-up in relevant interstitial lung sequelae (RILS) groups categorized by the Warrick score.

	**Non-RILS group (*n* = 198)**	**RILS group (*n* = 154)**	**Total (*N* = 352)**	^*^P-value
**PFT**				
FVC <80% pred., *N* (%)	25 (12.6)	54 (35.1)	79 (22.4)	<0.001
FEV_1_ <80% pred., *N* (%)	28 (14.1)	47 (30.5)	75 (21.3)	<0.001
DLCO <80% pred., *N* (%)	102 (51.5)	132 (85.7)	234 (66.5)	<0.001
FVC% pred., mean (SD)	96.5 (16.7)	88.3 (19.6)	92.9 (18.4)	<0.001
FEV1% pred., mean (SD)	102.6 (66.0)	91.0 (20.9)	97.5 (51.8)	<0.001
DLCO% pred., mean (SD)	80.6 (16.9)	60.9 (17.5)	72.3 (19.7)	<0.001
**HRCT, *N*(%)**				
Ground glass opacities	92 (46.5)	152 (98.7)	244 (69.3)	<0.001
Consolidations	3 (1.5)	30 (19.5)	33 (9.4)	<0.001
Irregular pleura	7 (3.5)	81 (52.6)	88 (25.0)	<0.001
Septal/subpleural lines	76 (38.4)	153 (99.4)	229 (65.1)	<0.001
Subpleural cysts	0 (0)	18 (11.7)	18 (5.1)	<0.001
Honeycomb	0 (0)	4 (2.6)	4 (1.1)	0.036
Architecture distortion	0 (0)	47 (30.5)	47 (13.4)	<0.001
Traction bronchiectasis	0 (0)	26 (16.9)	26 (7.4)	<0.001
Atelectasis	7 (3.5)	20 (13)	27 (7.7)	0.001
Nodules	10 (5.1)	18 (11.7)	28 (8.0)	0.022
Pleural effusion	0 (0)	4 (2.6)	4 (1.1)	0.036
Hypoattenuation	24 (12.1)	64 (41.6)	88 (25.0)	<0.001

* *P-value comparisons between non-RILS and RILS groups*.

*RILS, relevant interstitial lung sequelae defined as a Warrick score ≥ 7; DLCO, lung diffusing capacity for carbon monoxide; FEV_1_, forced expiratory volume in the first second; FVC, forced vital capacity; HRCT, high-resolution computed tomography; IQR, interquartile range; PFT, pulmonary function test; RILS, relevant interstitial lung sequelae*.

### Lung Ultrasound

The LUS findings of 352 patients at follow-up evaluations are shown in Table 5. Pleural line thickening in any area was found in 190 (53.9%) patients, and among them, only 12 presented with pleural line fragmentation. Pleural line thickening was significantly higher in the RILS group, and all patients with pleural line fragmentation were in this group. Only 3 patients showed pleural effusion and only 13 showed consolidations. In assessments of B-lines, 257 (73.0%) patients presented with pathological B-lines in any area. Among them, 52 (14.8%) patients presented coalescent B-lines, without significant differences between the RILS and non-RILS groups. Posterior-inferior areas showed a higher frequency of pathological B-lines.

**Table 5:** LUS findings at follow-up in relevant interstitial lung sequelae (RILS) groups categorized according to the Warrick score.

	**Non-RILS group (*n* = 198)**	**RILS group (*n* = 154)**	**Total (*N* = 352)**	^*^P-value
**LUS findings, *n* (%)**				
B-lines in any area	105 (53.0)	152 (98.7)	257 (73.0)	<0.001
B-lines ≥ 3 areas	36 (18.2)	145 (94.2)	181 (51.4)	<0.001
Coalescent B-lines	24 (12.1)	28 (18.2)	52 (14.8)	0.322
Thickened pleural line in any area	71 (35.9)	119 (77.3)	190 (53.9)	<0.001
Fragmented pleural line in any area	0 (0)	12 (7.8)	12 (3.4)	0.015
Pleural effusion	0 (0)	3 (1.9)	3 (0.9)	0.722
Consolidations	3 (1.5)	10 (6.5)	13 (3.7)	0.081
**LUS B-line score (0–12)**, median (IQR)	1.0 (0.0–2.0)	5.0 (4.0–9.0)	3.0 (0.0–5.0)	<0.001

* *P-value comparisons between non-RILS and RILS groups*.

*RILS, relevant interstitial lung sequelae defined as by Warrick score ≥7*.

*IQR, interquartile range; RILS, relevant interstitial lung sequelae*.

**Figure 3:**
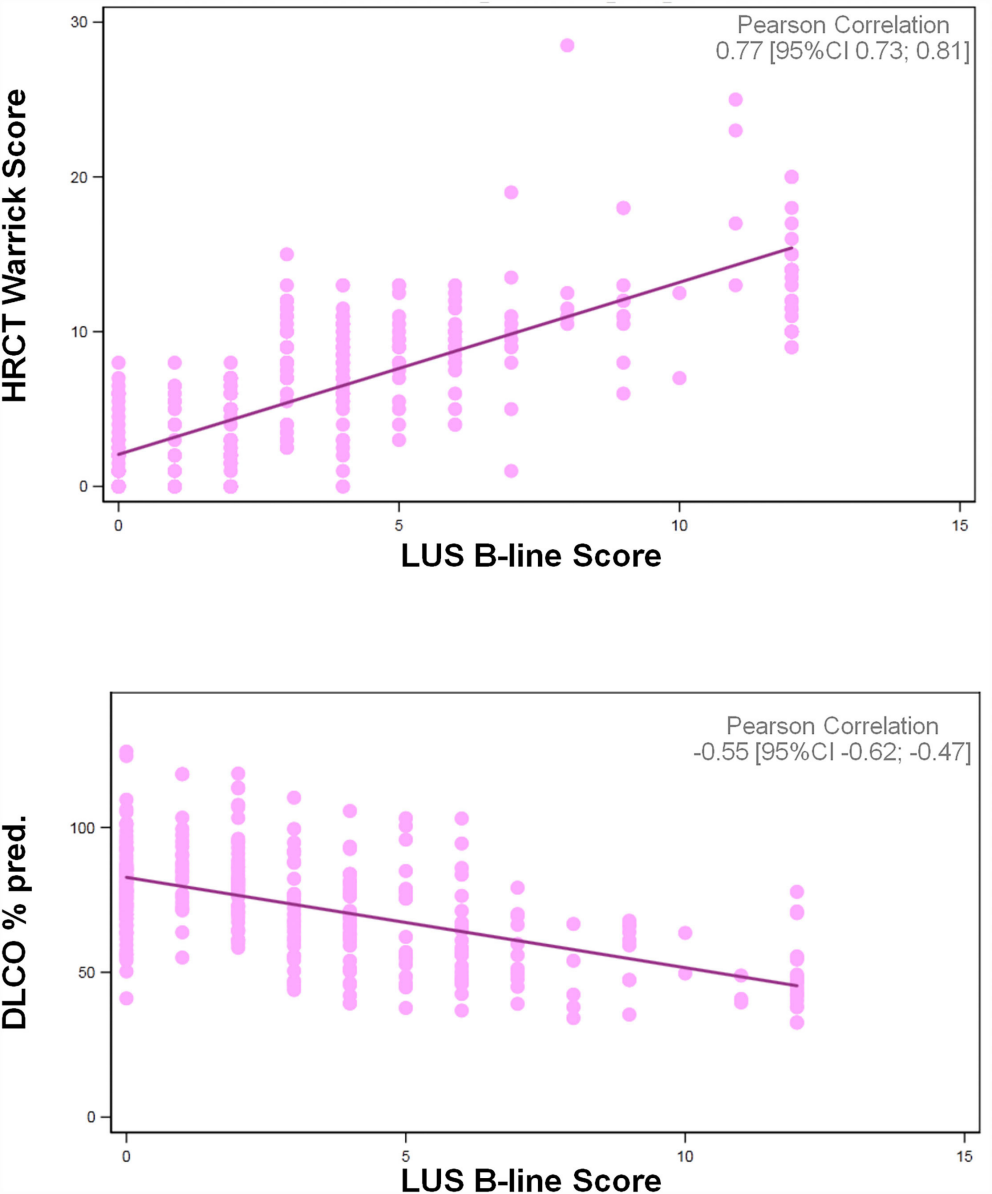
Correlations of LUS B-line score with HRCT Warrick Score and DLCO%pred.

The median (IQR) B-line score was significantly higher in patients in the RILS group (5.0 [4.0–9.0] vs. 1.0 [0.0–2.0], *p* < 0.001). The B-line score was strongly correlated with the HRCT Warrick score (r = 0.77) and showed a moderate inverse correlation with DLCO (r = −0.55) (Figure 3).

**Figure 4:**
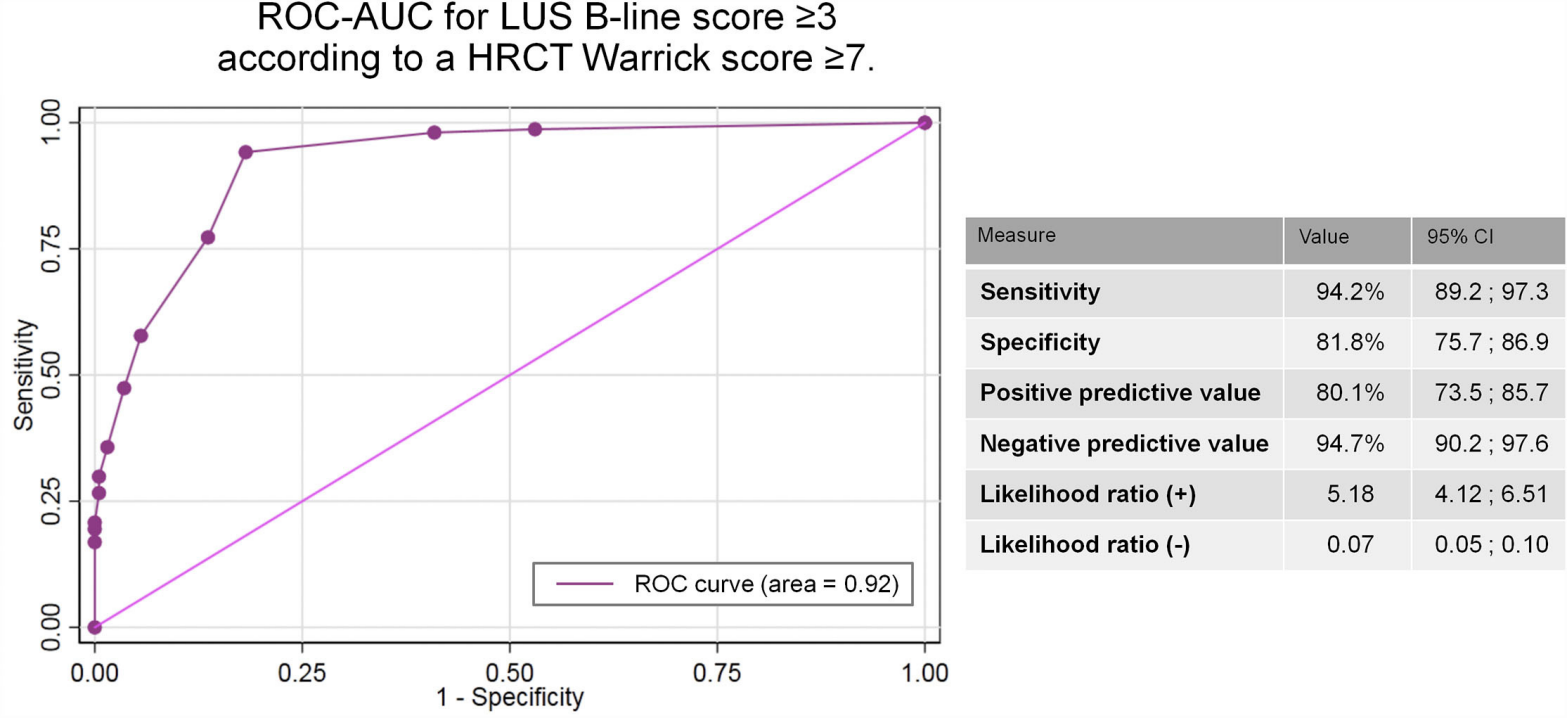
AUC-ROC to determine the LUS B-line score ability to discriminate patients with relevant interstitial lung sequelae after COVID-19 according to a HRCT Warrick score ≥ 7.

The ROC curve analysis showed that a B-line score of 3 or more was the best cut-off point to discriminate patients with RILS (Warrick score ≥ 7) (Figure 4). This value represented the best compromise between sensitivity (94.16%) and specificity (81.82%). The corresponding negative and positive predictive values were 94.74 and 80.11%, respectively, and the AUC was 0.92.

The B-line score showed excellent inter-observer agreement with a kappa value of 0.90 (95% CI 0.86–0.94). Detection of pleural-line abnormalities showed a poor inter-observer agreement, with a kappa index of 0.48 (95% CI 0.41–0.56).

## Discussion

To our knowledge, this is the first large study focusing on the role of LUS for the evaluation of early interstitial sequelae in COVID-19 survivors. The presence of pathological B-lines showed excellent ability to discriminate these persistent abnormalities in comparison with HRCT, supporting the use of LUS as a first-line procedure to rule out post-COVID-19 lung sequelae.

We report the follow-up findings for 352 patients evaluated 2–5 months after hospitalization for COVID-19 pneumonia. A high prevalence of functional and radiological sequelae was observed in our population, likely due to the high proportion of severe pneumonia cases. Indeed, data from larger series support the same prevalence rates of persistent radiological abnormalities (11–15), including fibrotic changes, in almost 35% of the severe cases (13). Similarly, lung function impairment, particularly decreased DLCO, has also been widely reported (22 to 82% of patients) and seems to be related to the severity of the pneumonia episode (14–16).

The natural history of COVID-19 lung sequelae is still unknown, and it is unclear whether full recovery can be expected in all cases. In addition, long COVID is another clinical entity that adds complexity to these uncertainties, as affected patients can manifest persistent respiratory symptoms without evidence of radiological or functional abnormalities on usual explorations (45, 46). As this new condition requires a complete investigation of potential pulmonary and extra-pulmonary complications, the presence of pulmonary sequelae should be ruled out.

Therefore, effective diagnostic tests are required for a correct identification and monitoring of patients with post-COVID-19 interstitial lung sequelae. Considering the immensely large population affected, an easy, low-cost, and reproducible diagnostic procedure is crucial to manage this clinical challenge. The belief that LUS could be useful for this purpose motivated the present study.

To date, only one report of 38 patients has described the use of LUS for detection of COVID-19 lung sequelae compared to HRCT (47). In this study, LUS results showed a high agreement rate compared to HRCT, but also a high proportion of false-negative findings in patients with milder forms.

In our study, 352 patients underwent complete LUS exploration of all intercostal spaces, and the findings were quantified in a 12-area score. We searched for the presence of a “lung interstitial syndrome” pattern, which was defined by the presence of multiple B-lines (44). These artifacts have very low specificity since they are present in different diseases, but they have high sensitivity, particularly in the detection of ILD (32–34). Various methods have been proposed for the evaluation of this pattern, and the reliability of these methods remains a topic of debate (48, 49). Quantification of the total number of B-lines has been extensively used (32–34); however, some authors have reported relevant variabilities among transducers and raters which could cause an important bias (50, 51). Semi-quantitative methods based on visual estimation of the percentage of space occupied by B-lines have also been proposed (52), but they probably lead to similar disadvantages. The application of traditional aeration LUS scores is also controversial in ILD, as the grade of deaireation has not proved to be able to discriminate between different degrees of interstitial involvement (53).

In our report, we implemented a simplified method since our goal was to discriminate patients with potential interstitial lung sequelae after COVID-19 and not to evaluate the grade or type of abnormalities. Based on the definition of the ICC-LUS (44), we considered pathological areas as those with more than two B-lines, and each area was scored 1 point out of a total of 12 thoracic areas. This score was strongly correlated with the HRCT Warrick score and showed excellent inter-observer reliability. Furthermore, a score ≥ 3 showed high sensitivity and specificity values (94.2 and 81.8%, respectively) and a negative predictive value of 94.7%. These solid results suggest that normal LUS examinations could rule out the presence of ILD in COVID-19 survivors, avoiding the need for additional diagnostic tests such as HRCT.

Pleural line abnormalities such as thickening and fragmentation have also been described as useful sonographic signs for the diagnosis of ILD. Various studies in the last decade have shown that pleural line measurements could be particularly accurate in ruling out pulmonary fibrosis (34, 35, 53). Pleural line abnormalities were frequent in our cohort, especially in the RILS group. However, we did not perform a pleural line measurement; therefore, our evaluation was subjective and derived on a poor inter-observer reliability. For these reasons, we did not include this finding in our score nor did we attempt to evaluate its diagnostic accuracy. Further studies are required to evaluate the significant pleural alterations identified by LUS in these patients.

Early identification of persistent post-COVID-19 interstitial sequelae seems to be crucial, particularly since it may facilitate prompt initiation of treatments to prevent permanent fibrotic changes. Furthermore, given the increasing number of cases worldwide, a diagnostic test available for large populations is essential. The technical characteristics of LUS seem suitable for this purpose since sonography is low-cost, non-ionizing, repeatable, and reproducible in outpatient clinics. Moreover, expertise and skills in LUS can be obtained with a short training trajectory (54), and the technique can be implemented in primary care to reach larger populations. Our report suggests that LUS yields high diagnostic accuracy for the detection of post-COVID-19 early sequelae. In particular, its high sensitivity and negative predictive values support its use as a first-line procedure to rule out these abnormalities in a potentially large population of patients. LUS implementation as a first-line examination could reduce the use of HRCT and help select patients who require more intensive follow-up, thereby improving the efficiency of health care assistance.

### Strengths and Limitations

The present study has some remarkable strengths. First, it is the largest prospective cohort published for this objective. Patients were recruited consecutively, and the same complete protocol was performed in a short time period. HRCT images, considered as the gold-standard, were evaluated by two independent thoracic radiologists, and in cases with significant differences, both radiologists met and performed a third consensual assessment. In addition, LUS operators were blinded to the clinical and radiological data and achieved excellent interobserver agreement. Finally, this was a reproducible and real-life study, since LUS was performed in the outpatient clinic by pulmonologists.

Yet, as in any study, there are some limitations. The order of follow-up visits was based on disease severity, potentially causing a relevant bias in the descriptive analysis. Consequently, the study population included a low percentage of mild cases, since many patients in this group had still not visited for follow-up assessments when we stopped study recruitment. However, none of these limitations, which are typical of a real-life study, influenced the primary objective. Another limitation is that we used the Warrick score, which has been validated in scleroderma-related ILD, to grade HRCT abnormalities because of the absence of any other specific score. Moreover, a Warrick cut-off point of ≥7 could lead to the underdiagnosis of milder forms of ILD. In our favor, it is likely that minor abnormalities are not clinically relevant in COVID-19 sequelae, since they could represent an intermediate step toward full recovery. In addition, this score has been widely applied in previous LUS reports of other ILDs and includes the main interstitial abnormalities found in COVID-19 survivors. Nevertheless, more studies are needed to describe how these lesions will evolve over time and whether LUS will continue to play a role in their detection.

## Conclusions

LUS could be implemented as a first-line procedure in the evaluation of interstitial lung sequelae after COVID-19 pneumonia. The identification of pathological B-lines in a twelve-area score showed a high negative predictive value for the detection of these abnormalities. Consequently, a normal LUS examination could rule out the presence of ILD in COVID-19 survivors, avoiding the need for additional diagnostic tests such as HRCT.

## Data Availability Statement

The datasets presented in this article are not readily available because approval from the relevant authorities is required. Requests to access the datasets should be directed to dclofent@vhebron.net.

## Ethics Statement

The studies involving human participants were reviewed and approved by Medical Ethics Committee of Vall d'Hebron Barcelona University Hospital [PR(AG)461/2020]. The patients/participants provided their written informed consent to participate in this study.

## Author Contributions

DC is the guarantor of the paper and takes full responsibility for the content of the manuscript, including data and analysis. DC, MC, and EP conceived and designed the study, analyzed the data, and drafted the manuscript. Statistical analysis was performed by Santiago Pérez-Hoyos. DC, AF, GG, and MA performed lung ultrasound examinations in all patients. JA, AS, DV, LC, and JE conducted the chest HRCT examinations in all patients. XM, AA, and KL critically reviewed the manuscript for relevant intellectual content. All authors approved the final version of the manuscript.

## Conflict of Interest

The authors declare that the research was conducted in the absence of any commercial or financial relationships that could be construed as a potential conflict of interest.

## Publisher's Note

All claims expressed in this article are solely those of the authors and do not necessarily represent those of their affiliated organizations, or those of the publisher, the editors and the reviewers. Any product that may be evaluated in this article, or claim that may be made by its manufacturer, is not guaranteed or endorsed by the publisher.

## Abbreviations

ARDS: acute respiratory syndrome
AUC: area under the curve
BMI: body mass index
COVID-19: coronavirus disease 2019
CT: computed tomography
CXR: chest radiography
DLCO: carbon monoxide diffusing capacity
FEV_1_: forced expiratory volume in the first second
FVC: forced vital capacity
GGO: ground-glass opacity
HFO: high-flow oxygen
HRCT: high resolution computed tomography
ICU: intensive care unit
IQR: inter-quartile range
ILD: interstitial lung disease
IMV: invasive mechanical ventilation
LFO: low-flow oxygen
LUS: lung ultrasound
MERS: middle east respiratory syndrome coronavirus
NIMV: non-invasive mechanical ventilation
PF: pulmonary fibrosis
PFT: pulmonary function tests
RILS: relevant interstitial lung sequelae
SARS-CoV: severe acute respiratory syndrome coronavirus
SARS-CoV-2: severe acute respiratory syndrome coronavirus 2
SD: standard deviation.
